# Lime plaster cover of the dead 12,000 years ago – new evidence for the origins of lime plaster technology

**DOI:** 10.1017/ehs.2019.9

**Published:** 2019-09-30

**Authors:** David E. Friesem, Itay Abadi, Dana Shaham, Leore Grosman

**Affiliations:** 1 McDonald Institute for Archaeological Research, University of Cambridge, Downing Street, Cambridge CB2 3ER, UK; 2 Zinman Institute of Archaeology, University of Haifa, 199 Aba-Hushi Avenue, 3498838 Haifa, Israel; 3 Mount Scopus, The Hebrew University of Jerusalem, Institute of Archaeology, 9190501 Jerusalem, Israel; 4 The Jack, Joseph and Morton Scholion–Mandel School for Advanced Studies in the Humanities, The Hebrew University of Jerusalem, 9190501 Jerusalem, Israel

**Keywords:** Lime plaster, Natufian, burial, pyrotechnology, southern Levant

## Abstract

The production of lime plaster is especially important as a technological development in human prehistory as it requires advanced knowledge and skills to transform rocks to a plastic yet durable material. The large-scale production of lime plaster is considered a development of farming societies during the Neolithic period around 10,000 years ago. To date, the archaeological evidence from the Middle and Late Epipalaeolithic in the southern Levant (c. 17,000–11,500 cal BP) indicates that only initial production of partially carbonated lime plaster was performed by Palaeolithic foragers. Our study analysed lime plaster covering burials at a Natufian cemetery in Nahal Ein Gev II, dating to 12,000 years ago. Using infrared spectroscopy and soil micromorphology we show how this lime plaster is of an unprecedented high quality and we reconstruct its production. The results exhibit a technological leap forward at the end of the Palaeolithic. We provide a new model for understanding the evolutionary paths of lime plaster technology during the Palaeolithic–Neolithic transition.

## Graphical abstract

**Figure:**
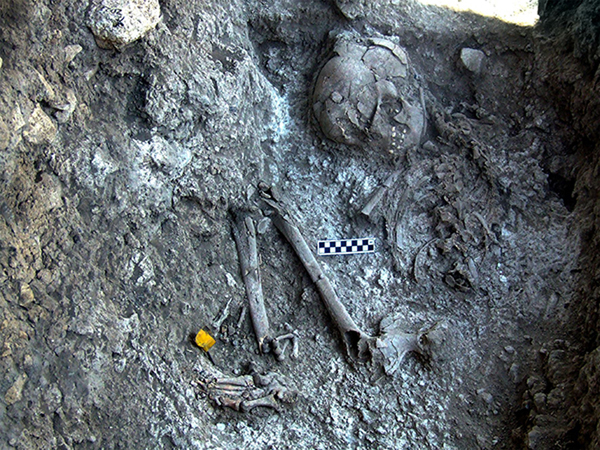

**Social media summary:** Lime plaster covering burials 12,000 years ago presents a technological leap forward at the end of the Palaeolithic

## Introduction

The knowledge and skill to produce synthetic materials using fire is one of several important trajectories in the technological evolution of human history. Fire can be used as a tool to transform materials into new materials with new properties (e.g. cooking food, making pottery out of clay and making durable plaster from rocks). The production of lime plaster involves different stages of preparation that must be carried out carefully in order to produce a high-quality product. First, quicklime powder is produced by burning rocks (commonly limestone, chalk and marl) at very high temperatures (>700°C). The lime is then mixed with water to form a plastic putty that can be applied and shaped. Upon drying, the plaster retains durable properties and exhibits a hard surface. Technological developments in the making of lime plaster included the addition of different materials to give the plaster different properties, for example better binding properties or resistance to water (Boynton 1980). Thus, the technology behind lime plaster production necessitates intricate knowledge and skills.

This kind of material manipulation on a massive scale and the widespread production of lime plaster are considered a development of farming societies during the Pre-Pottery Neolithic B (PPNB), which took place in the Levant beginning about 10,000 years ago (Clarke 2012 and references therein; Gourdin and Kingery 1975; Kingery *et al.* 1988). Although there is sporadic evidence of small-scale production of synthetic materials used as adhesives from the Middle Palaeolithic and even slightly earlier (Mazza *et al.* 2006; Pawlik and Thissen 2011; Wadley *et al.* 2009), early evidence for production of quicklime and lime plaster dates back to the Middle and Late Epipalaeolithic in the southern Levant (Bar-Yosef and Goring-Morris 1977; Goring-Morris *et al.* 1997; Kingery *et al.* 1988; Valla *et al.* 2007). Compared with the well-studied lime plaster technology during the PPNB (Chu *et al.* 2008; Clarke 2012; Goren and Goring-Morris 2008; Goren and Goldberg 1991; Goren *et al.* 2001; Goring-Morris and Horwitz 2007; Gourdin and Kingery 1975; Kingery *et al.* 1988; Poduska *et al.* 2012; Regev *et al.* 2010a; Toffolo *et al.* 2017), studies of lime plaster associated with Epipalaeolithic foragers is to date very limited (Chu *et al.* 2008; Kingery *et al.* 1988; Valla *et al.* 2007).

**Figure 1.**
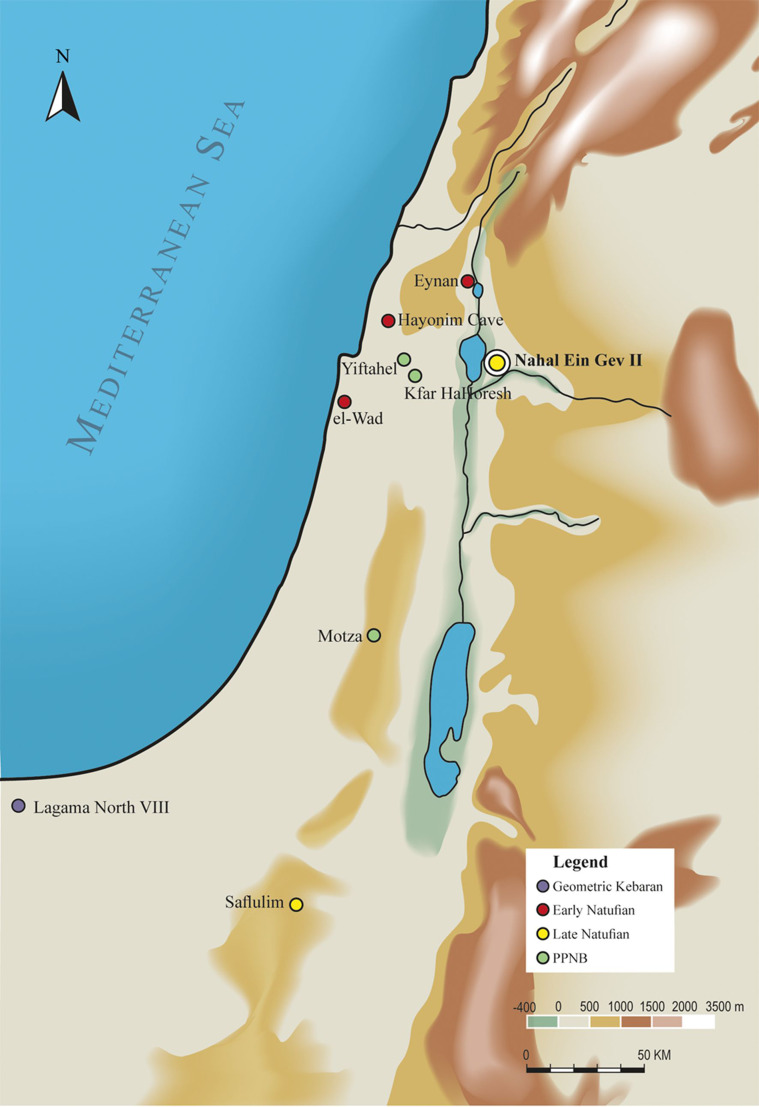
Map of the southern Levant showing the location of Nahal Ein Gev II (NEG II) and other Epipalaeolithic and Neolithic sites mentioned in the text.

**Figure 2.**
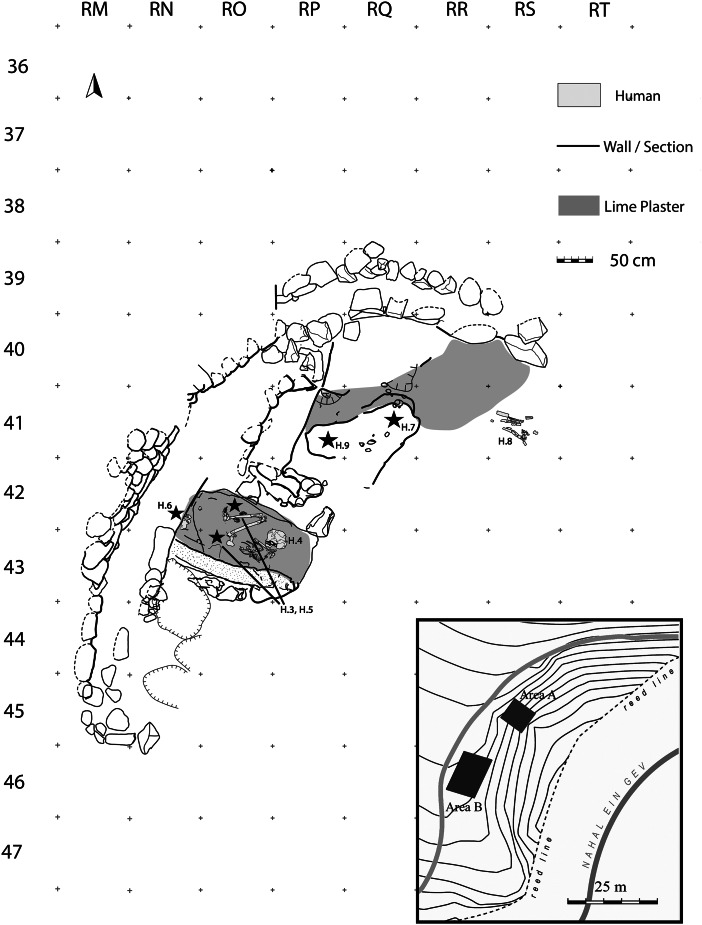
Plan of the NEG II with enlarged map of area A showing the coverage of the white plaster layer and location of human remains.

At the Late Epipalaeolithic site Nahal Ein-Gev II (NEG II), located at the Upper Jordan Valley (Figure 1), a cemetery dated to 12 thousand (calibrated) years before present [k cal BP] (Grosman *et al.* 2016) was unearthed revealing, to date, eight individuals covered by a ca. 40 cm thick layer of a white dense material (Figure 2; see details below) interpreted in the field as lime plaster. This exceptional finding in an Epipalaeolithic context called for an in-depth analysis of the white material covering the burials in order to understand its position within the evolutionary paths of lime plaster technology during the Palaeolithic–Neolithic transition. Thus, the aim of our study was first to identify and characterize the white material at microscopic and chemical scale using infrared spectroscopy and micromorphological analysis. Based on the material properties revealed in the laboratory by the geoarchaeological analysis, we were able to understand how the plaster at NEG II was formed, and to reconstruct its production technology. The results of our analysis not only confirm the identification of the white material covering the burials as a pyrogenic lime plaster, but also provide new evidence for large-scale production of high-quality lime plaster at the end of the Epipalaeolithic, a technology previously associated with the PPNB c. 2000 years later. Framing our study within the existing archaeological evidence of Palaeolithic and Early Neolithic lime plaster technology, we offer a new model for the technological evolution of lime plaster.

### Geoarchaeological background

The production of lime plaster requires the exploitation of rocks composed mainly of calcium carbonate (CaCO_3_ – usually found in the form of calcite mineral) which if burnt at very high temperatures (>700°C) for a prolonged time, usually using kilns, transform into calcium oxide (CaO – also termed quicklime). Quicklime is slaked with water, forming a putty of calcium hydroxide (Ca[OH_2_]) to obtain plasticity. At this stage a wide range of materials can be added to the slaked lime to obtain various characteristics (e.g. quartz [sand], ashes, dung, vegetal matter, ceramic and volcanic ash). The slaked lime putty is then shaped and applied, and as the material dries and reacts with the air in the atmosphere it transforms back to calcium carbonate (Boynton 1980). From a chemical perspective, this production technique is known as the lime cycle because it involves the transformation of calcium carbonate from a geological origin into calcium oxide and calcium hydroxide before transforming back into calcium carbonate in the form of pyrogenic calcite. While geogenic calcium carbonate is characterized by atomic ordered calcite, the rapid formation of pyrogenic calcium carbonate results in a microcrystalline and highly atomic disordered calcite (Chu *et al.* 2008; Kingery *et al.* 1988; Poduska *et al.* 2011; Regev *et al.* 2010a; Shoval *et al.* 2003; Shoval *et al.* 2011; Weiner 2010). Thus, the atomic order/disorder of calcite serves as an important indicator for the formation processes of the calcite and as a reliable proxy for the pyrotechnology involved in the production of calcite-based plaster materials (Chu *et al.* 2008; Goshen *et al.* 2017; Poduska *et al.* 2012; Regev *et al.* 2010a, b; Toffolo *et al.* 2017). In addition to mineralogical analysis of lime plaster, observations of the plaster under the microscope can reveal the quality of the plaster, by examining the extent of carbonation of the plaster matrix, and identifying different additive materials (Goren and Goring-Morris 2008; Goren and Goldberg 1991; Goren *et al.* 2001; Goshen *et al.* 2017; Karkanas 2007; Kingery *et al.* 1988; Poduska *et al.* 2012; Toffolo *et al.* 2017). Overall, geoarchaeological methods, in particular mineralogical analysis using infrared spectroscopy and micromorphological analysis of thin sections, have proven to be highly useful for investigating lime plaster technology in the past (Weiner 2010, p. 190).

### Prehistoric lime plaster in the southern Levant

To date, the earliest evidence for the use of burnt lime in the Levant was reported from Lagama North VIII in the Sinai, where remnants of burnt lime were found on the back of trapeze-rectangle microliths and interpreted as an adhesive material for hafting during the Geometric Kebaran (c.16 k cal BP) (Bar-Yosef and Goring-Morris 1977). Scanning electron microscope (SEM) and energy dispersive X-ray analysis (EDX) suggested that the material is composed of a pure and microcrystalline calcium carbonate interpreted as lime plaster (Kingery *et al.* 1988). Similar evidence for the use of a limestone-based adhesive material on a sickle was also reported from the Natufian assemblages in el-Wad on Mt Carmel (Tomenchuk 1984). Micromorphological analysis of similar adhesive material from the Late Natufian (c. 13 k cal BP) site of Saflulim in the Negev (Goring-Morris *et al.* 1997) demonstrated that the material is composed of microcrystalline calcium carbonate and contains several layers, reflecting multiple events of coating.

Lime plaster was reported from Early Natufian burials at Eynan (‘Ein Mallaha) (14.7–13 k cal BP; Chu *et al.* 2008; Valla *et al.* 2007). Infrared analysis of flat white pieces from the graves suggested that it is lime plaster composed of calcite that is slightly more disordered that geological rocks, but not as disordered as experimental lime plaster (Chu *et al.* 2008; Valla *et al.* 2007). SEM/EDX analysis of a hard material bench found at the site (Perrot 1966, 1975) suggested that it is a partly carbonated lime plaster mixed with a small amount of aluminosilicate (Kingery *et al.* 1988).

A hearth interpreted as a combustion feature for quicklime production (Bar-Yosef 1983; Kingery *et al.* 1988) was found at Hayonim Cave, dating to the Early Natufian (c. 14 k cal BP) (Chu *et al.* 2008). A 20 cm thick white porous layer was found within the hearth. SEM/EDX analysis showed it to be composed of calcium carbonate, which was interpreted as partly carbonated lime plaster (Kingery *et al.* 1988). Infrared analysis indicated that the calcite in the hearth was slightly disordered (Chu *et al.* 2008). As in the case of the white material from the burials in Eynan, the calcite in the hearth in Hayonim Cave was more disordered then limestone but not as expected to be in a well-carbonated lime plaster.

In Saflulim, a plastered floor surface, analysed via micromorphology, was reported to exhibit cemented domains and mixing with cultural deposits (e.g. charcoal and organic matter) and natural deposits. The mixed nature of the matrix suggested the presence of partly carbonated lime plaster, slaked with archaeological sediment and the local loess (Goring-Morris *et al.* 1997).

Later, during the PPNB, extensive use of lime plaster and the diversification of its technology are considered as some of the identifying characteristics of this period (Goren and Goldberg 1991; Goren and Goring-Morris 2008; Goren *et al.* 2001; Gourdin and Kingery 1975; Karkanas 2007; Kingery *et al.* 1988). Yet only a few studies have examined PPNB lime plaster in the southern Levant through microscopic and chemical analysis. PPNB lime plaster is in most cases characterized by a partly carbonated and impure matrix exhibiting mixing of the lime with local sediments and anthropogenic materials (e.g. charcoal, ash, bones, dung, flint, plants, etc.; Goren and Goldberg 1991; Goren *et al.* 2001; Karkanas 2007; Kingery *et al.* 1988). Cases of pure lime plaster displaying a well-carbonated matrix are rarer but not uncommon (Goren and Goldberg 1991; Goren *et al.* 2001; Kingery *et al.* 1988; Poduska *et al.* 2012). Mineralogical analysis of PPNB lime plaster showed that the calcite atomic order among PPNB lime plasters is usually slightly disordered, being borderline between pyrogenic formation and disordered geogenic formation, as in chalk (Chu *et al.* 2008; Poduska *et al.* 2012; Regev *et al.* 2010a; Toffolo *et al.* 2017). Some PPNB plasters have been argued to have been produced without the use of fire by pulverizing chalk and mixing it with water and clay before applying it on surfaces (Goren and Goldberg 1991; Friesem *et al.* 2014).

In terms of the preservation of prehistoric lime plaster, the calcitic microcrystals may undergo diagenesis in which the calcite atomic disorder will tend towards a more ordered structure (Weiner 2010, p. 190). However, most prehistoric lime plaster that shows other indications for well-carbonated lime plaster also displays calcite atomic disorder associated with pyrogenic formation (Chu *et al.* 2008; Poduska *et al.* 2012; Regev *et al.* 2010a; Toffolo *et al.* 2017). In Yiftahel, a highly disordered calcite was found in a PPNB plastered floor alongside ordered calcite formed through diagenetic processes (Poduska *et al.* 2012). On the one hand, this evidence demonstrates how diagenesis can occur in well-carbonated lime plaster, resulting in taphonomic transition towards ordered calcite, but on the other hand it shows that traces of highly disordered calcite were still trackable in the weathered plaster (Poduska *et al.* 2012). Overall, PPNB lime plaster manufacture in the southern Levant represents a complex technology involving the admixture of a wide range of materials that allowed manipulation of the plaster material in different ways while retaining a high-quality standard (Clarke 2012; Goren and Goldberg 1991; Goren *et al.* 2001; Malinowski 2011).

### Nahal Ein Gev II

In order to follow the cultural, economic and technological characteristics during the Palaeolithic–Neolithic transition, we study the Late Natufian site NEG II dated to 12.5–12 k cal BP (Grosman *et al.* 2016). The Natufian culture is recognized as important during the dynamic turnover in human evolution to food-producing cultures in the southern Levant. It is of special interest not only because of significant cultural changes that emerged as human populations became more permanently settled, but also because it set the stage for the fundamental transformation to agriculturally based societies (Grosman and Munro 2017). The increase in site permanence, site size and human population size across the transition to agriculture required the re-organization of communities to accommodate associated social changes (Goring-Morris and Belfer-Cohen 2008; Munro and Grosman 2018). Many important trajectories of social change that begin in the Natufian, including permanent settlement infrastructure, heightened symbolic communication and ritual practice, and the concentration burial of human remains in settlements, extend into the Neolithic period. The resulting changes reflect the increasingly differentiated use of space and a rise in communal architecture, as well as new site functions and activities (Grosman and Munro 2016; Liu *et al.* 2018; Richter *et al.* 2016; Rosenberg and Nadel 2014; Valla 2018).

**Figure 3.**
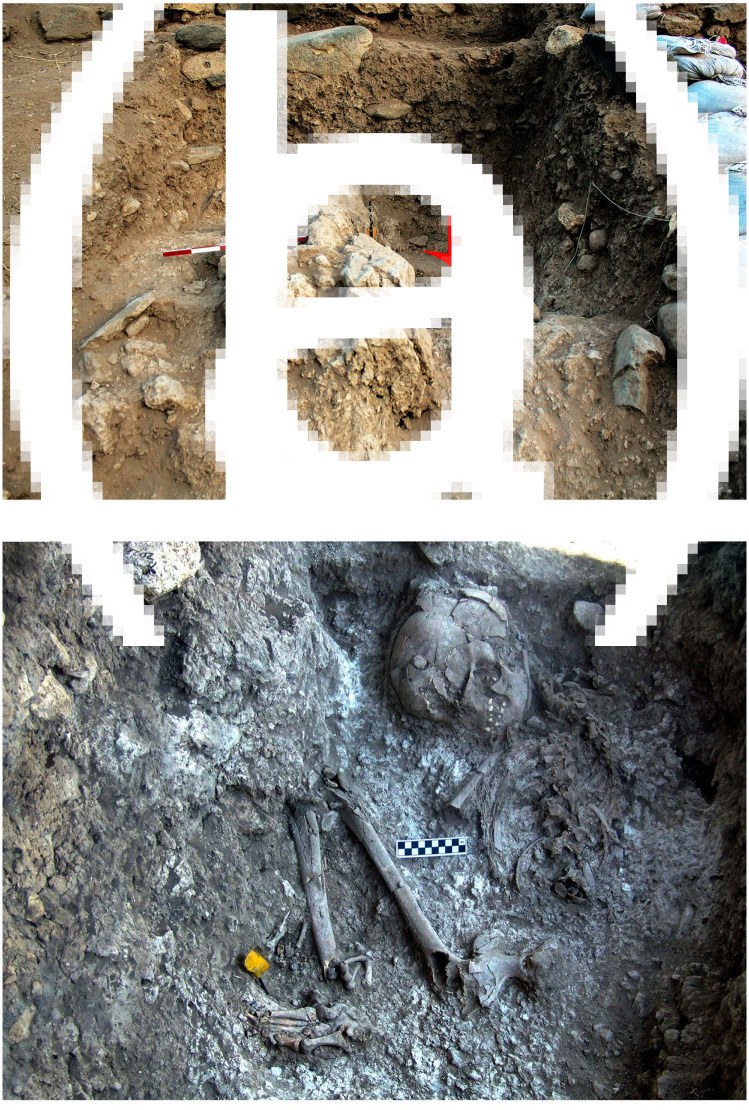
The massive white material layer. (a) Field photograph showing the volume of the white layer with the location of a human burial H.4 (arrow). (b) Close-up of a human burial (H.4) embedded in the white material.

NEG II is located in the wadi of Ein Gev, flowing west into the Sea of Galilee (Figure 1). Renewed excavations at NEG II started in 2010 (Israel Antiquities Authority Permits G-57/2010, G-49/2011, G-73/2012, G-3 76/2013, G-78/2015, G-77/2016). Field work was carried out in the eroded section facing east (ca. 540 m long) of the site at two excavation areas, A and B. The characteristics of the material remains from both areas are homogeneous to a depth of more than ca. 3 m, suggesting occupation by a single cultural entity, the Late Natufian (Grosman *et al.* 2016). The excavations in Area A were divided into two stages. The first explored the stratigraphic nature of the site in a trench (2 × 3 m) starting from the 1 m high wall located on the upper surface of the eroded slope. The trench was excavated as a series of steps along the erosional slope, exposing the entire stratigraphic sequence of the site reaching a massive white material arround and under a human burial, Homo 4 [H.4] (Figure 3). In 2012 a grid system (1 × 1 m squares) was put in place across a 25 m^2^ in Area A for the excavation of a larger horizontal surface on both sides of the trench (Figure 2). Each square metre was further divided into four sub-squares (50 × 50 cm). A locus number was provided for each unique unit of excavation following the nature of sediment or stratigraphic observations (maximum unit depth is 5 cm). Each unique feature or special item was plotted with three coordinates using a total station theodolite.

**Figure 4.**
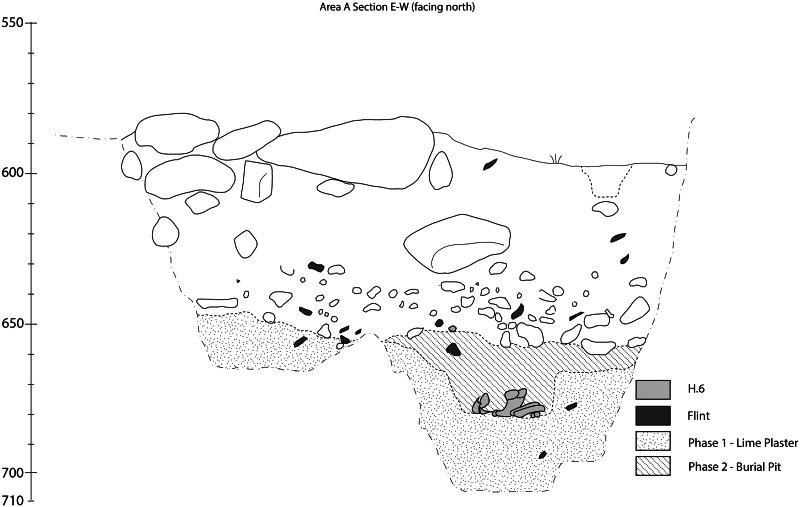
A cross-section (east–west) displaying the stratigraphic relations of the different archaeological units.

The top of the burial ground was reached 1 m below the base of a five-course wall which was sealed by a distinct surface of small stones (Figure 4). The extent and depth of the burial ground is still to be determined. Within the initial trench we reached human remains of four individuals (H.3, H.4, H.5 and H.6, Figure 2). A female skeleton was resting at the base of the trench (H.4) in a completely articulated, flexed position (Figures 2 and 3b). The hard white sediment around her skeleton and the smoothed plaster under her skull suggest that her body was covered with lime plaster at the time of burial. The bones of two other individuals (H.3, H.5) were scattered around H.4. In the section of the trench, an additional articulated burial (H.6) was detected (Figure 4). In the extended area of excavation, beyond the trench, the top of the lime plaster layer was recently reached. Only a small portion of the layer has been removed thus far, yet near the surface the remains of two skulls were exposed (H.7, H.9). In addition, scattered bones of an additional skeleton (H.8) were observed on the eroded white surface of the eastern edge of the burial ground (Figure 2). The surface of the lime plaster is still to be peeled in order to expose the complete skeleton remains at the burial ground. Three distinct stratigraphic phases (from base to top) were observed in the trench (Figure 4):

1. The first phase was the caliche bedrock dug into by the Natufians for the interment of the dead and covered with the white material. So far, this caliche bedrock was observed in the burial pit of H.4 at the trench. However in other areas of the burial ground, bedrock was not reached.
2. The second phase was the interment of burials in pits dug into the white layer then covered with the local sediment – a mixture of the cultural deposits, regional sediment and bulks of the white material (H.6).
3. Finally, both phases were sealed with a layer of small stones.

Recent excavation defined the boundaries of the plaster layer that marks the extent of the burial area that reaches a monumental wall to the North of the original trench (Figure 2), but the boundaries and extent of the burial ground and the lime plaster covering it to the south are still unclear, as well as its depth.

## Materials and methods

### Fourier-transform infrared spectroscopy

Samples of loose bulk sediment and rocks (*n* = 17) were collected from Area A according to sediment colour, texture and hardness. Samples were collected from: the massive white layer covering the burials (*n* = 7); mixed sediments from the white layer and above it, ranging from grey to brown in colour (*n* = 4); sediments infilling the later pits dug into the massive white layer (*n* = 5 sediments and *n* = 1 stone found inside the sediments); and control rock samples (*n* = 11) from the vicinity of the site including limestone, chalk, marl and dolomite ranging from the Middle Eocene to Miocene (Michelson 1972).

The samples were analysed using Fourier-transform infrared (FTIR) spectroscopy in order to identify the major mineral components for each sample (Weiner 2010, p. 275). The spectra were collected using the KBr method between 4000 and 400 cm^−1^, at 4 cm^−1^ resolution and interpreted using an internal library of infrared spectra of archaeological materials (Weiner 2010, pp. 276–277). To evaluate the atomic order/disorder in calcite mineral resulting from its formation processes (e.g. geological, biological or pyrogenic), the ν_2_ and ν_4_ absorption bands, corresponding to 874 and 713 cm^−1^ respectively, were studied by calculation of their height ratio (ν_2_/ν_4_; Chu *et al.* 2008). Alongside the ν ratio method of Chu *et al.* (2008), we also applied the grinding curves method of Regev *et al.* (2010b). The latter involves repeated grinding and analysis of the same sample, producing several spectra of this sample, showing different extents of grinding. Grinding curves were obtained for each sample by plotting the changes in each spectrum in the ν_2_ and ν_4_ absorption bands height, normalized to the height of the ν_3_ absorption band at 1415–1440 cm^−1^, reported in normalized absorbance units (n.a.u.) (Regev *et al.* 2010a). In addition, the full width of the calcite ν_3_ absorbance band, at 1415–1440 cm^−1^, was calculated at half of the band height, reported as full width half maximum, as a measure for the extent of grinding (Chu *et al.* 2008; Poduska *et al.* 2011; Regev *et al.* 2010a). Evaluation of clay alteration owing to exposure to high temperatures (>500°C) was based on the presence/absence of absorption bands at 915, 3625 and 3695 cm^−1^ (Berna *et al.* 2007).

### Soil and sediment micromorphology

Petrographic thin sections (*n* = 6) for micromorphological analysis were prepared from impregnated undisturbed monolithic sediment blocks (*n* = 3) sampling the sedimentary sequence from above the white layer (sample 14-2) to its bottom (sample 14-3). In addition, a thin section was made from off-site sediments (sample 14-6) and used as a control sample.

The undisturbed monolithic sediment blocks were sampled using Plaster-of-Paris jackets and were prepared for micromorphological analysis following conventional procedures (Courty *et al.* 1989). The blocks were dried in an oven at 50°C for 3 days and then impregnated using a 9:1 mixture of polyester resin and acetone and 1% v/v MEKP. Pre-cut sample slices (50 × 76 mm) were sent to Quality Thin Sections, Tuscon, Arizona, where 30 µm thin sections were prepared. Thin sections were first studied at a scale of 1:1, scanned using a flatbed scanner and then analysed with petrographic microscopes at magnifications ranging from ×4 to ×400 with plane-polarized light (PPL) and cross-polarized light. Micromorphological descriptions employ the terminology of Stoops (2003).

## Results

### Mineralogical analysis

**Figure 5.**
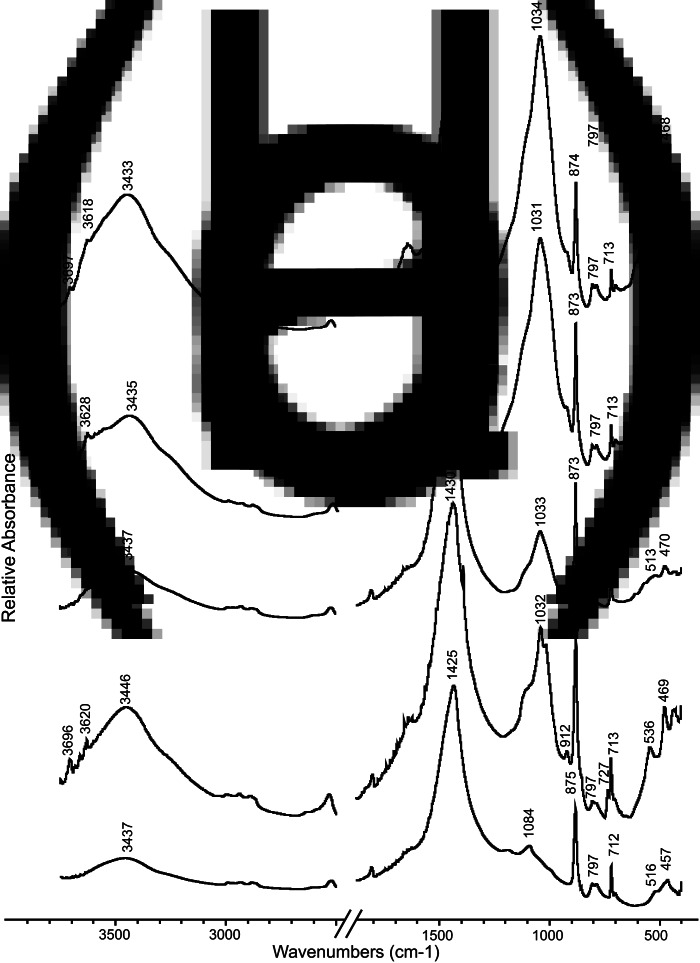
Representative Fourier-transform infrared (FTIR) spectra of samples from NEG II. (a) Control limestone sample (C-8) from the site vicinity. Note the indicative calcite absorption band located at 1425, 875 and 712 cm^−1^. The sample also contains a small amount of quartz (3437, 1084, 797, 516 and 457 cm^−1^). (b) Control marl sample (C-3) from the site vicinity. Note how alongside calcite as the major mineral, there are minor components in form of unaltered clay (3446, 3620, 3696, 1032, 912, 797, 536 and 469 cm^−1^) and dolomite (727 cm^−1^). (c) The white material (16-3) representing pyrogenic lime plaster with indicative absorption band for highly disordered calcite (1436 cm^−1^ and the high ratio between the height of 873 and 713 cm^−1^). Note the very low amount of clay and quartz (3437, shoulders at 1084, 1033, 513 and 470 cm^−1^) with the former showing alteration owing to exposure to elevated temperatures, indicated by the absence of absorption bands at 915, 3625 and 3695 cm^−1^. (d) Brownish sample (16-6) representing mixed anthropogenic sediment with lime plaster (16-6) composed of unaltered clay (3697, 3628, 3435, 1031, 797, 513 and 468 cm^−1^) mixed with disordered calcite. (e) Grey sediment from a pit (16-9) showing unaltered clay and ordered calcite.

**Figure 6.**
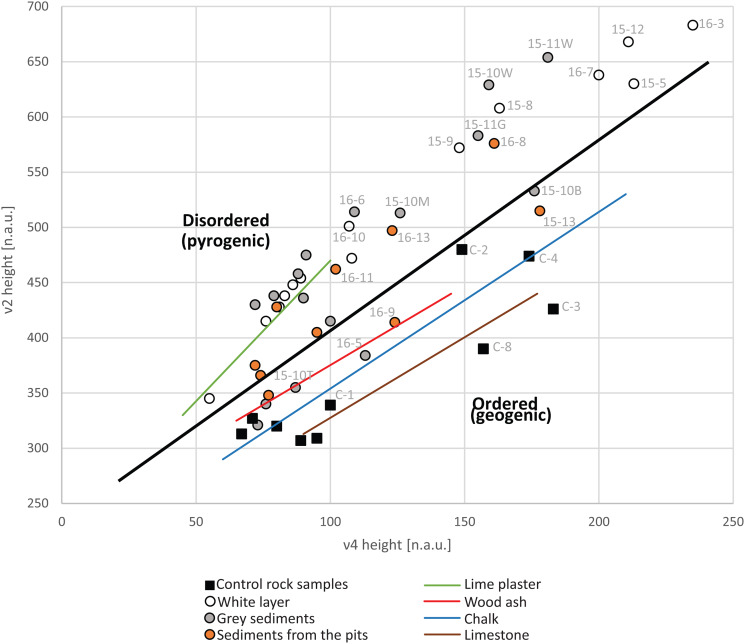
FTIR spectroscopy analysis of calcite atomic order/disorder. The chart shows values of the ν_2_ and ν_4_ infrared absorbance band in normalized absorbance units (n.a.u.) following sequential grinding of regional control rock samples (squares) and archaeological samples (circles) in relation to experimental data by Regev *et al.* (2010a) (coloured solid lines). The black solid line marks the division between geogenic and pyrogenic formation of calcite. Each sample is presented by the two end points of the grinding curve with the name of the sample appearing at the point marking the first measurement.

Results of FTIR analysis of bulk sediment and rock samples provided evidence for the mineralogical composition and formation processes of the samples (Table 1). The mineralogical composition of the regional control rock samples showed either dolomite or calcite as the major mineral component. Among the rocks composed mainly of calcite (*n* = 5, Table 1), some of the limestone showed minor amounts of quartz, dolomite and apatite (Figure 5a). The marl displayed, alongside calcite as the major component, clay as well as dolomite as minor mineralogical components (Figure 5b). In all of the regional control rock samples the calcite atomic order is within the expected range of geogenic calcite (Regev *et al.* 2010a; Figure 6).

**Table 1.** Description of bulk sediments and rock samples and Fourier-transform infrared results

Sample	Context	Description	Major Minerals	*ν*_2_ (n.a.u.)	ν_4_ (n.a.u.)	Full width half maximum (cm^−1^)	Calcite atomic order	Clay pyrogenic alteration	Interpretation
15-5	RN 42 b, −6.62	White material	Ca >> Cl	630–448	213–86	185–112	Highly disordered	Altered	Pyrogenic lime plaster
15-8	RO 42 c, −6.78	White material	Ca, Cl	608–472	163–108	183–126	Disordered	Unaltered	Pyrogenic lime plaster mixed with unburnt clay
15-9	RO 42 d, −6.67	White material	Ca > Cl	572–415	148–76	154–110	Disordered	Unaltered	Pyrogenic lime plaster mixed with unburnt clay
15-10T	RO 42 b, −6.55	Grey material – top	Cl >> Ca	355–340	87–76	232–140	Ordered	Unaltered	Unburnt anthropogenic sediment
15-10M	RO 42 b, −6.55	Grey material – middle	Cl > Ca	513–436	126–90	159–120	Slightly disordered	Unaltered	Mixed anthropogenic sediment with lime plaster
15-10B	RO 42 b, −6.55	Grey material – bottom	Ca, Cl	533–415	176–100	162–114	Ordered	Unaltered	Unburnt anthropogenic sediment
15-10W	RO 42 b, −6.55	White piece in grey material	Ca >> Cl	629–430	159–72	154–105	Highly disordered	Altered	Piece of pyrogenic lime plaster
15-11G	RQ 41 a, −6.70	Grey and white material covering skull	Cl, Ca	583–438	155–79	162–110	Disordered	Unaltered	Mixed anthropogenic sediment with lime plaster
15-11W	RQ 41 a, −6.70	White piece in grey and white material covering skull	Ca >> Cl	654–475	181–91	169–116	Highly disordered	Altered	Piece of pyrogenic lime plaster
15-12	RQ 41 a, −6.63	White material	Ca >> Cl	668–454	211–89	187–115	Highly disordered	Altered	Pyrogenic lime plaster
15-13	RP 41 d, −6.84	Grey material from a pit	Cl, Ca	515–405	178–95	179–118	Ordered	Unaltered	Unburnt anthropogenic sediment
16-3	RP 43 c, −6.97	White material	Ca >> Cl	683–438	235–83	189–111	Highly disordered	Altered	Pyrogenic lime plaster
16-5	RO 42 b, −6.45	Grey material	Cl > Ca	384–321	113–73	152–115	Ordered	Unaltered	Mixed anthropogenic sediment with burnt clay
16-6	RO 42 b, −6.57	Brown yellowish material	Ca, Cl	514–458	109–88	143–133	Disordered	Unaltered	Mixed anthropogenic sediment with lime plaster
16-7	RO 42 d, −6.71	White material	Ca >> Cl	638–428	200–81	185–115	Highly disordered	Altered	Pyrogenic lime plaster
16-8	RP 42 c, −6.90	White brown material from a pit	Ca >> Cl	576–428	161–80	150–112	Disordered	Altered	Pyrogenic lime plaster
16-9	RN 43 d, −6.75	Grey material from pit, Locus 33	Ca, Cl	414–348	124–77	169–118	Ordered	Unaltered	Unburnt sediment
16-10	RQ 41 b, −6.72	White material	Ca >> Cl	501–345	107–55	132–95	Disordered	Altered	Pyrogenic lime plaster
16-11	RQ 41 b, −6.77	White and grey material from pit, Locus 14	Cl, Ca	462–366	102–74	144–115	Slightly disordered	Unaltered	Mixed anthropogenic sediment with lime plaster
16-12	RQ 41 b, −6.77	White stone from Locus 14	D >> Ca						Geogenic rock
16-13	RN 45 a, -6.57	White and grey material, Locus 38	Ca > Cl	497–375	123–72	149–106	Slightly disordered	Unaltered	Mixed anthropogenic sediment with lime plaster
C-1	Maresha Formation, member of Zor'a (Middle–Lower Eocene)	Limestone-chalk	Ca, Q > D	339–327	100–71	159–140	Ordered		Geogenic rock
C-2	Maresha/Fiq Formation (Upper Eocene–Oligocene)	Detrital limestone	Ca >> Ap	480–313	149–67	149–95	Ordered		Geogenic rock
C-3	Lower Susita Formation (Oligocene–Lower Miocene)	Marl	Ca > Cl > D	426–307	183–89	191–123	Ordered	Unaltered	Geogenic rock
C-4	Lower Susita Formation (Oligocene–Lower Miocene)	Marl	Ca >> D > Cl	474–320	174–80	170–101	Ordered	Unaltered	Geogenic rock
C-5	Suista Formation (Oligocene–Lower Miocene)	Dolomite	D > Cl					Unaltered	Geogenic rock
C-6	Susista Formation (Oligocene–Lower Miocene)	Dolomite rich in organic fossils	D						Geogenic rock
C-7	Upper Susita Formation (Oligocene–Lower Miocene)	Dolomite	D						Geogenic rock
C-8	Noqev Formation, member of Susita (Lower Miocene)	Limestone with quartzlite	Ca > Q	390–309	157–95	148–108	Ordered		Geogenic rock
C-9	Upper Susita fomration (Lower Miocene)	Dolomite above sample 8	D						Geogenic rock
C-10	Maresha Formation, member of Zor'a (Middle–Lower Eocene)	Dolomite	D						Geogenic rock
C-11	Fiq fomration (Upper Eocene–Oligocene)	Dolomite near the site	D						Geogenic rock

Minerals: Ca, calcite; Cl, clay; Q, quartz; D, dolomite; Ap, apatite. n.a.u., Normalized absorbance units.

The white material covering the burials (*n* = 7, Table 1) is composed mainly of calcite with clay being a minor component (Figure 5c). The extent of the calcite atomic disorder in the white material is typical for well-carbonated lime plaster, indicating its pyrogenic formation (Poduska *et al.* 2011; Regev *et al.* 2010a; Toffolo *et al.* 2017). Except for two samples (15-8 and 15-9), the clay component in the white layer seems to be altered by exposure to high temperatures (Berna *et al.* 2007) (Figure 5c, Table 1). Overall, the white material corresponds well with pyrogenic formation of lime plaster.

White and grey sediments found within the white layer (*n* = 3, Table 1), and one sample classified in the field as brown yellowish sediment (16-6, Figure 5d), were characterized by clay and calcite as the major components. These samples exhibited unaltered clay and calcite atomic order at the borderline between geogenic atomic order and pyrogenic atomic disorder (Figure 6). Although such values are also produced by wood ash (Regev *et al.* 2010a), given the context at the top of the white layer, it is more likely that these sediments represent admixture between lime plaster from the white layer and geogenic calcite found in the local sediment. White pieces found within these sediments showed highly disordered calcite and altered clay and were interpreted as fragments of the white material, further supporting the idea of mixing.

Sediment samples collected from the pits that were dug into the white layer (*n* = 6, Table 1) presented both calcite and clay as major mineralogical components (Figure 5e). The clay minerals were unaltered while the calcite atomic order showed a borderline disorder (Figure 6), suggesting either deposition of ash or mixing of the local unburnt sediment that infilled the pits with fragments of lime plaster into which the pits were dug.

### Micromorphology

**Figure 7.**
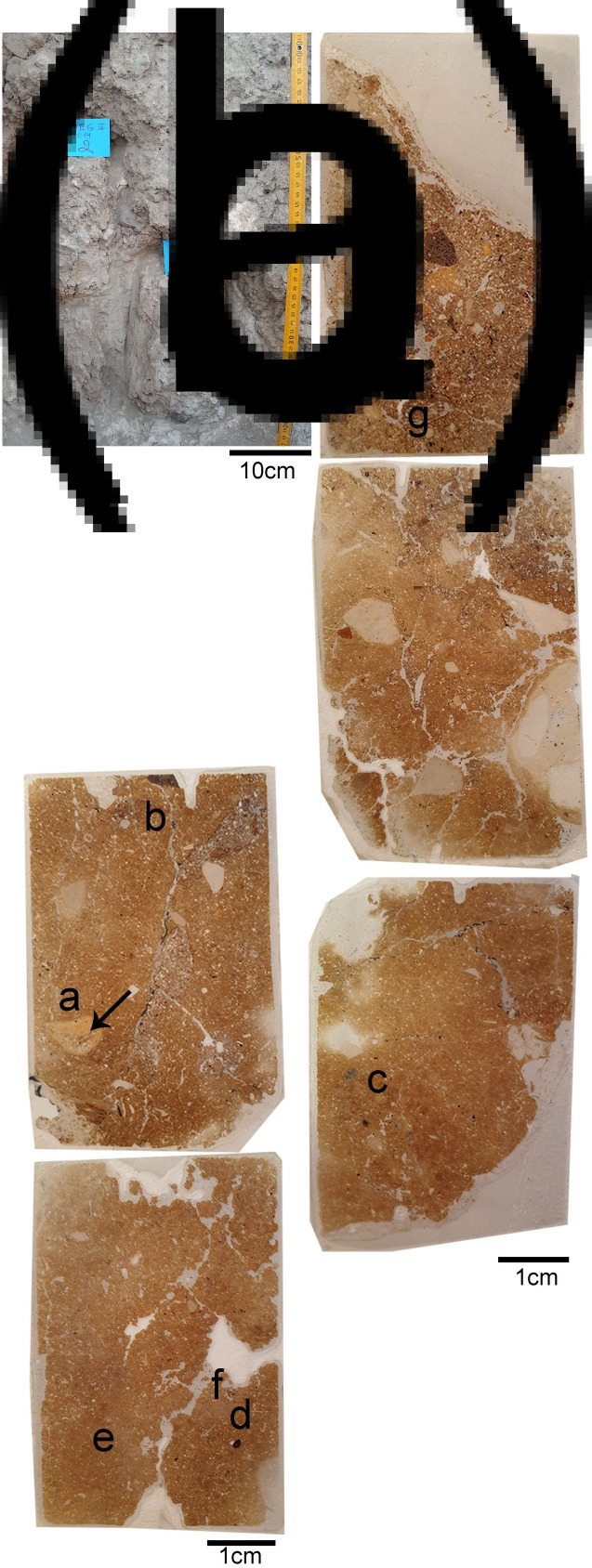
Thin sections from the white layer. (a) Field photograph of the western profile of sq. RO42 in Area A showing the location of two block sediment samples. Sample14-2 covers the upper part of white layer and the sediment above it. Sample 14-3 covers the lower part of the white layer just above the locations of burials. (b) Scan of thin sections from 14-2. (c) Scan of thin sections from 14-3. Note the compact structure of the white layer and the planar voids indicative to shrinkage fractures. The arrow marks a large bone fragment embedded in the white material. Letters mark the location of the microphotographs shown in Figure 8.

**Figure 8.**
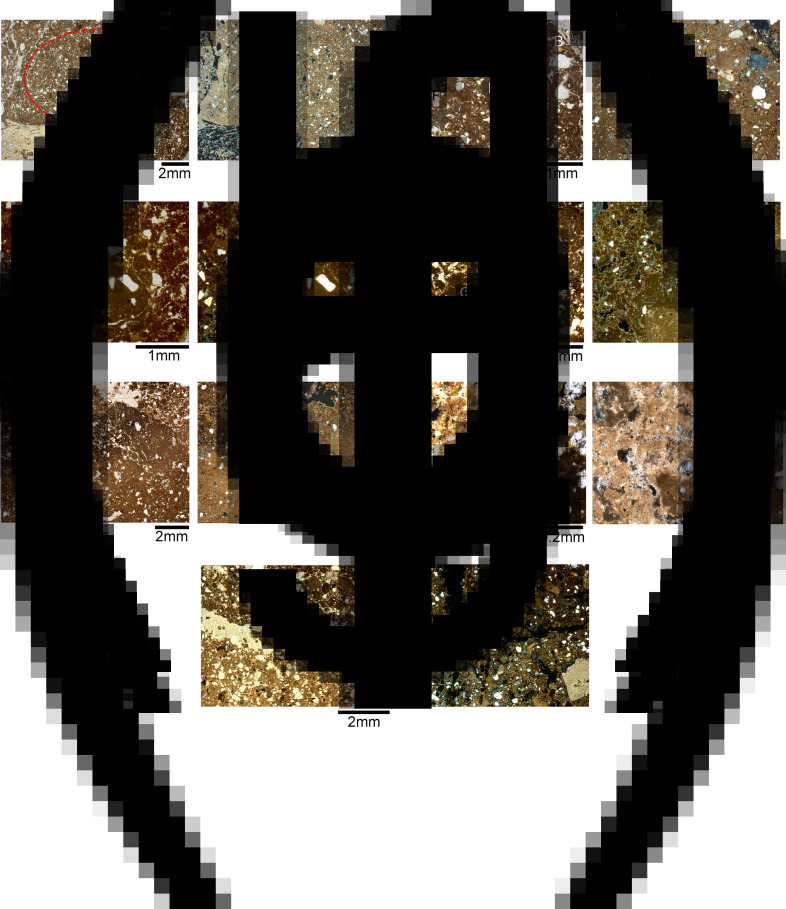
Micromorphology of the lime plaster. In all microphotographs the left image is in plane-polarized light (PPL) and the right image is in cross-polarized light. (a) Dense and moderately sorted matrix with a large bone fragment (B) and a rotated sedimentary feature (red dashed line) owing to the pugging activity. (b) A mixed matrix with poorly reacted limestone fragments (L) and shell (S), bone (B) and flint (F) fragments embedded in partly carbonated micritic groundmass with some clay. (c) A burnt limestone fragment (L) showing a reaction rim and quicklime attached to it reacting with the partly carbonated groundmass. Note the bone fragments (B) embedded in the cemented matrix. (d) Partly carbonated groundmass rich in quicklime showing burnt limestone fragments (L) and quicklime lumps (Q) poorly reacting with the groundmass. (e) Contact between a well-carbonated matrix (bottom) and a partly carbonated matrix rich in quicklime (top). (f) Close-up on the contact between the well- and partly carbonated areas. Note the yellowish colour and high birefringence of the well carbonated matrix, indicating that it fully reacted, and the pelletal structure of the dark grey quicklime with low birefringence, indicating only partial reaction. (g) The contact between the white layer (bottom), interpreted as lime plaster showing a dense and cemented calcitic groundmass, and the local archaeological sediment showing a poorly sorted calcitic-clay groundmass (top).

**Figure 9.**
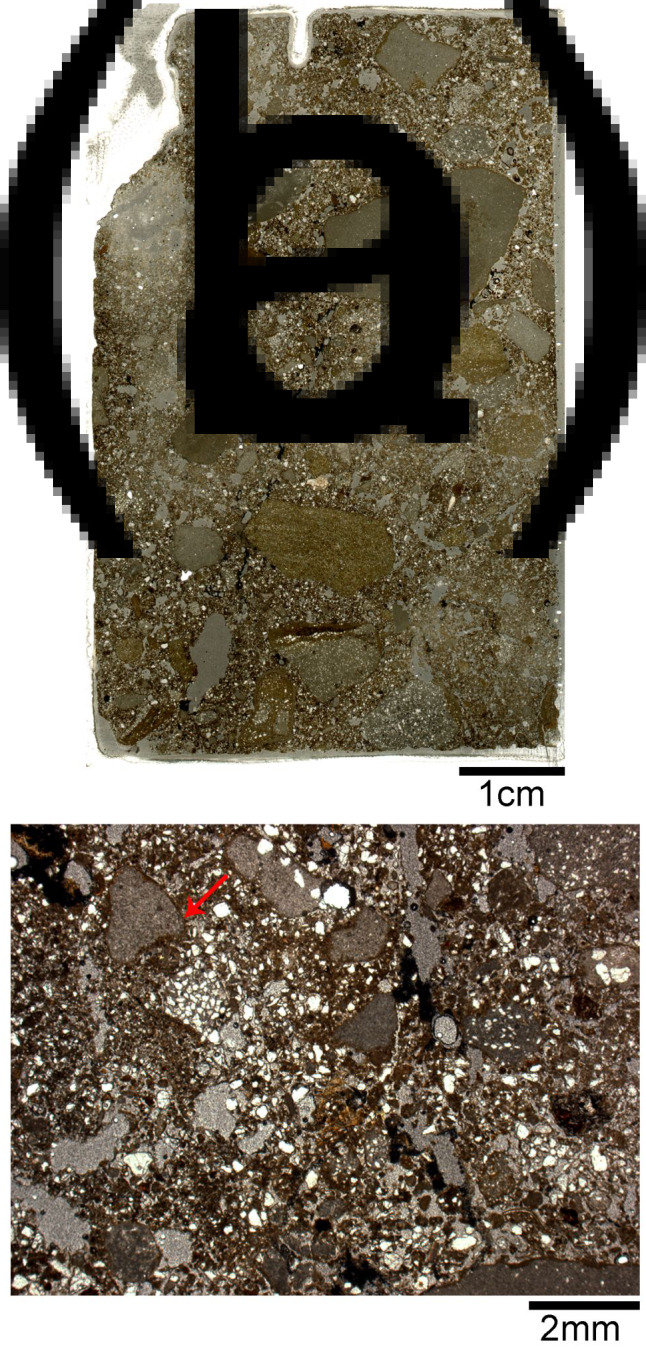
Micromorphology of the regional sediment. (a) Scan of a thin section from the off-site sediment sample (14-6). Note the presence of gravels in the clay-rich matrix. (b) Microphotograph showing a poorly sorted matrix with an open microstructure. Note the presence of unburnt limestone fragments covered by continues clay coating (arrow) typical for slope deposition. Image taken in PPL.

The thin sections produced from the white massive layer covering the burials displayed a compact structure with decreased porosity and elongated planar voids (Table 2, Figure 7). Such planar voids are common in lime plasters, indicating shrinkage fractures occurring as the plaster dries (Karkanas 2007). The matrix is moderately sorted with relatively homogenous distribution of the coarse fraction within the groundmass and pressure-induced deformation pedofeatures (e.g. shearing, rotation and directionality of flow; Figure 8a). These pedofeatures evince pugging and indicate the deliberate preparation of this material as opposed to unworked natural sediments (Friesem *et al.* 2014). The coarse fraction includes moderately sorted silt to sand size quartz, bones (some are burnt), shells and flint fragments (Figures 8a and b). In addition, the presence of burnt limestone fragments, some with reaction rims (Figure 8c) and quicklime lumps (Figure 8d), suggests that some of the parts of the raw material were not thoroughly burnt and/or fully reacted with the groundmass during slaking (Karkanas 2007). Under the microscope, the white layer presents two distinctive matrices (Figure 8e). Both matrices show a groundmass rich in microcrystalline calcite, but with some clay as well, cementing the coarse fraction. However, while the first exhibits a very well-carbonated and fully reacted matrix with high birefringence, the second is rich in quicklime with dark grey pelletal structure and low birefringence indicating only partial reaction and carbonation (Karkanas 2007; Figure 8f). The presence of well-carbonated matrix, quicklime lumps and limestones reacting with the groundmass supports the interpretation of the white layer as pyrogenic lime plaster (Goren and Goldberg 1991; Goren *et al.* 2001; Goshen *et al.* 2017; Karkanas 2007; Kingery *et al.* 1988; Poduska *et al.* 2012; Toffolo *et al.* 2017). None of these features was observed in the local sediment on- and off-site (Table 1, Figures 8g and 9).

**Table 2.** Micromorphological description and interpretation of thin sections

Sample	Location	Field description	Key micromorphological observations	Interpretation
14-6	Slope 40 m northeast to the site	Grey soft and crumbly sediment with pebbles and some fragments of flint	Poorly sorted fabric, heavily bioturbated and mixed Calcitic-clay groundmass with crystallitic birefringence fabric and a porphyric-related distribution Complex microstructure with channels, chambers, vesicles and voughs voids Coarse fraction includes moderately sorted silt to sand quartz (15%), fragments of limestone, shells, microcharcoal and bone and flint fragments (<5%) The grains of the coarse fraction show continuous clay coating indicative for slope deposition	Regional sediment
14-2A	Area A RO42 −6.41/−6.59	Grey crumbly sediment with a layer of small stones on top of the upper part of the white material layer	Poorly sorted fabric, with areas of pelletal aggregates, heavily bioturbated Calcitic-clay groundmass with crystallitic birefringence fabric and a porphyric-related distribution Complex microstructure with channels, chambers and vesicle voids Coarse fraction includes moderately sorted silt to sand size quartz (15%) microcharcoal, rock, shell, bone and flint fragments and organic matter (<5%)	Local archaeological sediment
14-2B/C		Massive white layer	Sorted, dense and well cemented matrix Groundmass rich in micritic calcite Compact structure, decreased porosity Planar voids indicative for shrinkage fractures Pressure-induced deformation pedofeatures due to pugging Some areas showing well-carbonated matrix Some areas showing moderately carbonated matrix rich in unreacted quicklime Coarse fraction includes moderately sorted silt to sand size quartz (15%), quicklime lumps, burnt limestone, bones (some are burnt), shells and flint fragments (<5%)	Lime plaster
14-3	Area A RO42 −6.56/−6.75

The local sediment sampled off-site is characterized by a calcitic-clay groundmass with crystallitic birefringence and a porphyric-related distribution. The matrix is poorly sorted and heavily bioturbated, showing a complex microstructure with channels, chambers and vesicle voids. The coarse fraction includes gravels, moderately sorted silt to sand size quartz, microcharcoal, shell, bone and flint fragments, and organic matter (Figure 9).

The white layer itself is overlain by a sediment very similar to the off-site regional sediment but with higher abundance of anthropogenic residues such as microcharcoal, shell, bone and flint fragments and organic matter. Thus, we interpret this sediment as the local archaeological deposit. The contact between the white layer and the local sediment is quite sharp (Figure 8g), indicating two episodes of deposition and the good state of preservation of the lime plaster.

## Discussion

### Reconstruction of lime plaster technology at NEG II

The unequivocal evidence for the presence of pyrogenic lime plaster at NEG II burial ground is based on the following features we identified in the white material covering the human remains: (1) the extent of calcite atomic disorder (Figure 6); (2) the compact structure and shrinkage fractures (Figure 7b and c); (3) signs for pugging (Figure 8a); and (4) the well-carbonated and cemented microcrystalline groundmass (Figure 8e–g). Beyond the mere identification of lime plaster at NEG II cemetery, we use our data to suggest a reconstruction of the technology involved in its production.

The first step in the production of the lime plaster involved the acquisition of raw material in the form of local rocks rich in calcite. The area around the site presents access to several geological formations ranging from the Middle Eocene to the Miocene (Michelson 1972) exposing a wide range of rocks including limestone, chalk, marl and dolomite (Table 1). The presence of clay in the lime plaster material, alongside calcite as the major component, could suggest either the use of local marl as the raw material or the mixing of with the local sediment. However, the marl from the site's vicinity showed minor amounts of dolomite (Figure 5b). While dolomite can transform into calcite at 500°C (Goren *et al.* 2004; Weiner 2010, p. 204), it is more likely to leave traces of magnesium (Weiner 2010, pp. 188, 193), which were not found in any of the samples from the white layer (Table 1). Since dolomite is abundant in the site's vicinity, but also has poor binding properties (Weiner 2010, p. 188), we conclude that the Natufians in NEG II deliberately chose limestone or chalk, over the wide range of carbonate rocks available in their immediate environment, while being aware of its properties to produce high-quality lime plaster.

The next step would have been to burn the raw material in order to produce quicklime. Based on the extent of the calcite atomic disorder (Figure 6) and the alteration of the clay (Figure 5c) in the samples from the white layer covering the bodies, we suggest that it was exposed to very high temperature (>700°C). The presence of half-burnt limestone points out that some parts of the raw material were not burnt thoroughly, eliminating the possibility of prolonged use of kilns, as required to achieve complete transformation of all of the calcium carbonate in the raw material into calcium oxide (i.e. quicklime). Based on experimental studies that examined the production of lime plaster in prehistory, we suggest that the Natufians in NEG II used either open fires, probably with pulverized rocks (Karkanas 2007), or pit kilns that have been shown to preserve poorly in the archaeological record (Goren and Goring-Morris 2008). Nevertheless, the extensive area covered by lime plaster evinces the use of a large quantity of raw material exposed to very high temperatures for at least a few hours (Goren and Goring-Morris 2008; Karkanas 2007). Since the burial ground in NEG II is still being excavated, we cannot at this stage offer estimates for the amount of raw material and fuel used to produce this lime plaster as we are yet to unearth its full extent.

It has been argued that many Neolithic lime plasters in the Near East were produced by ‘hot mixing’ (Karkanas 2007). As opposed to the preparation of a lime putty in advance (by thoroughly slaking quicklime with water) and then applying the putty on a surface, in ‘hot mixing’, quicklime is being mixed with water *in situ* while hot and in many cases also with sediment. Thus, ‘hot mixing’ commonly results in heterogeneous slaking of the quicklime, leaving lumps and unreacted areas (Karkanas 2007). The micromorphology of the lime plaster in NEG II showed some areas to be very well carbonated and fully reacted while other parts of the lime plaster are only partly carbonated and rich in unreacted quicklime, as shown by the presence of quicklime lumps and pelletal structure in the groundmass (Figure 8d–f). Thus, we suggest that in NEG II quicklime and half-burnt limestone were mixed with water and the local archaeological sediment (containing bone, shell and flint fragments). This mixing took place on the burials themselves while the quicklime was still hot enough to burn some of the bones in the local sediment and alter the clay at >500°C. The mixing in this technique was probably uneven, resulting in some clays in the plaster layer remaining unaltered (Table 1) and in unreacted and poorly slaked areas (Figure 8d–f).

In the absence of pottery, the question of how exactly people transferred quicklime – known as an extremely hazardous material causing irritation to the skin, eyes and respiratory system – from the kilns or hearths onto the burial ground, remains an open question. It is possible that they used either skin, wood or rock containers that simply did not preserve in the archaeological record, but to date, no evidence has been found to support any of these possibilities.

During later phases in the activity at NEG II, pits were dug into the lime plaster for more burials to be placed within the thick lime plaster layer. Mineralogical analysis of the materials infilling the pits showed that the sediments in the pits display geogenic calcite (Figure 6) and unaltered clay (Figure 5e), but some white materials found within the pits’ sediments showed slightly disordered calcite and altered clay (Figure 6, Table 1). Thus, we interpret the pits to be filled with unburnt local sediment mixed with fragments of the lime plaster the pits were dug into.

Lastly, the sharp boundary between the lime plaster and the local sediment overlaying it (Figure 8g) suggests that the sediment on top of the plaster did not react with the lime, and thus was probably deposited some time after the plaster had cooled down and dried. Unfortunately, based on micromorphological evidence alone, we cannot estimate exactly how much time after the formation of the lime plaster this sediment was deposited.

### The evolutionary paths of lime plaster technology

**Figure 10.**
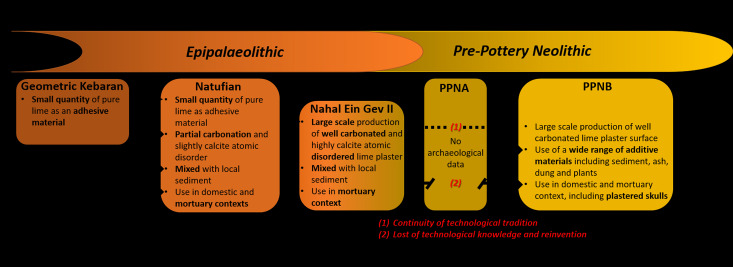
A suggested model for the evolutionary paths of lime plaster technology during the Epipalaeolithic and Pre-Pottery Neolithic in the southern Levant.

Recent geoarchaeological studies have demonstrated that mineralogical analysis, and in particular evaluation of calcite atomic disorder, integrated with micromorphological observations is the best method to evaluate lime plaster technology and quality (Goshen *et al.* 2017; Poduska *et al.* 2012; Regev *et al.* 2010b; Toffolo *et al.* 2017). We therefore applied a comparison of geoarchaeological data (Table 3) to offer a new model for the evolutionary paths of lime plaster technology during the Palaeolithic–Neolithic transition in the southern Levant (Figure 10). Unfortunately, to date the number of studies that applied such methods is quite limited. Yet based on the currently available geoarchaeological data from Epipalaeolithic and PPNB sites in the southern Levant, we argue that the lime plaster in NEG II should be regarded as a technological leap forward, marking an important evolutionary step in lime plaster technology.

**Table 3.** Results of geoarchaeological analysis of lime plasters from Epipalaeolithic and representative Pre-Pottery Neolithic B (PPNB) sites in the southern Levant.

Period	Site	Description	Calibrated date (ka)	*ν* Ratio (*ν*_2_/*ν*_4_)*	ν_2_/ν_4_ grinding curves	Microscopic observations	References
Modern	Experimental	Lime plaster		5.8–7.2	Highly disordered	Well-carbonated matrix	Karkanas 2007; Regev et al., 2010a
PPNB	Yiftahel	Surfaces	9–10	ND	Partly highly disordered and partly ordered	Well-carbonated matrix with indications for diagenesis and re-precipitation of calcite	Regev et al., 2010a; Poduska et al., 2012
	Kfar Hahoresh	Surfaces	~9	4.0–4.4	ND	Partly carbonated matrix mixed with clay	Arpin 2004; Chu et al., 2008; Goren and Goring-Morris 2008
	Motza	Surfaces	~10	3.5–4.0	ND	ND	Chu et al., 2008
	Nesher Ramla quarry	Lime kiln	10.4	ND	Slightly disordered	Mixed sediment showing exposure to high temperatures, use of plant fuel and lime fragments	Toffolo et al., 2017
Natufian	**Nahal Ein-Gev II**	**Thick layer covering burials**	**12**	**4.6–5.8**	**Highly disordered**	**Well-carbonated and partly carbonated matrix mixed with local sediment**	
	Saflulim	Surfaces	11?	ND	ND	Moderately carbonated lime mixed with anthropogenic materials and local loess	Goring-Morris et al., 1997
		Adhesive material on microlith		ND	ND	Small quantity of lime plaster	
	el-Wad	Adhesive material on microlith		ND	ND	Small quantity of lime plaster	Tomenchuk, 1984
	Hayonim Cave	Hearth	~14	3.8–4.1	ND	Partly carbonated lime plaster	Bar-Yosef, 1983; Kingery et al., 1988; Chu et al., 2008
	Eynan	Bench	15.3–12.9	ND	ND	Partly carbonated lime plaster with aluminosilicates	Perrot, 1966, 1975; Kingery et al., 1988; Valla et al., 2007; Chu et al., 2008;
		Flat pieces from grave		3.3–4.3	ND	Partly carbonated lime plaster with indications for diagenesis	
Geometric Kebaran	Lagama North VIII	Adhesive material on microlith	~16	ND	ND	Small quantity of pure lime plaster	Bar-Yosef and Goring-Morris, 1977; Kingery et al., 1988

All the results, except for NEG II (marked in italic and bold), are taken from previous studies, see reference list.

ND, No data.

* *ν* ratio is calculated on FTIR spectra with *ν*_3_ full width half maximum between 130 and 110 (Chu *et al.* 2008).

In terms of production technique, early experiments with burning lime are evident during the Middle Epipalaeolithic, when pure lime was produced in very small quantities for hafting lithics as an adhesive material (Bar-Yosef and Goring-Morris 1977; Kingery *et al.* 1988). This technology seems to continue into the Natufian (Goring-Morris *et al.* 1997; Tomenchuk 1984), but with the development of new ways of producing and exploiting lime.

During the Early Natufian period, lime plaster was produced in small scales for plastering surfaces and in burials, as evident from Eynan (Kingery *et al.* 1988; Perrot 1964 1975; Valla *et al.* 2007). Analysis of calcite atomic disorder of Natufian lime plasters showed it to be only slightly disordered (Chu *et al.* 2008; Valla *et al.* 2007) and partly carbonated (Goring-Morris *et al.* 1997; Kingery *et al.* 1988). Natufian lime plasters from a bench in Eynan (Kingery *et al.* 1988) and a plastered floor from Saflulim (Goring-Morris *et al.* 1997) were reported to contain some aluminosilicate and traces of local loess with anthropogenic activity residues, respectively, indicating the mixing of quicklime with the local sediment.

In many aspects, the lime plaster from NEG II seems to continue earlier Natufian technology, not only in its use in mortuary context but also in its production technique. The presence of half-burnt limestone in NEG II plaster suggests that burning of the raw material did not take place in a closed kiln for several hours or even days. Rather, it was probably burnt using an open hearth, such as the one found in the Natufian layers of Hayonim Cave dating to c. 14 k cal BP (Chu *et al.* 2008; Kingery *et al.* 1988), or in a pit kilns as reported from PPNB sites (Goren and Goring-Morris 2008; Toffolo *et al.* 2017). Owing to the poor preservations of open hearths and pit kilns in the archaeological record (Goren and Goring-Morris 2008), it is impossible to determine whether the evidence from NEG II represents a technological innovation or continuity in that aspect.

The lime plaster in NEG II shows mixing of quicklime with the local sediment containing anthropogenic activity residues, similar to the reports from Natufian plasters (Goring-Morris *et al.* 1997; Kingery *et al.* 1988), but with no evidence for other additive materials as commonly found in PPNB plasters (e.g. Goren and Goldberg 1991; Goren *et al.* 2001; Toffolo *et al.* 2017). However, NEG II lime plaster exhibits unprecedented properties compared with other Natufian lime plasters, including the extent of calcite atomic disorder, the carbonation of the matrix and its volume (Table 3). Thus, we conclude that the lime plaster at the NEG II burial ground took Natufian lime plaster technology into a larger scale of production and higher quality.

When compared with PPNB lime plasters, NEG II presents a higher extent of calcite atomic disorder even from most PPNB plasters (Chu *et al.* 2008; Poduska *et al.* 2012; Regev *et al.* 2010a; Toffolo *et al.* 2017), except the one reported from Yiftahel (Poduska *et al.* 2012). This indicates not only its high quality but also its remarkable preservation, probably owing to its exceptional volume as opposed to thinner plaster surfaces common during the PPNB (see Poduska *et al.* 2012 for diagenesis of a well-carbonated PPNB plastered floor). Overall, PPNB lime plaster technology appears as a direct continuation of the lime plaster in NEG II based on: the evident relation between lime plaster production and mortuary practices (Clarke 2012; Goren *et al.* 2001; Goring-Morris and Horwitz 2007; Kuijt and Goring-Morris 2002); large-scale production of mixed well-carbonated and partly carbonated matrix (Goren and Goldberg 1991; Goren *et al.* 2001; Kingery *et al.* 1988; Poduska *et al.* 2012); the ability to achieve high calcite atomic disorder (Poduska *et al.* 2012); and the use of ‘hot mixing’ technique (Goren *et al.* 2001; Karkanas and Goldberg 2007). Based on this evidence we argue that significant PPNB technological innovation lay in mastering the art of admixture between quicklime and a wide range of materials including clay, wood ash, dung and vegetal matter (Goren and Goldberg 1991; Goren *et al.* 2001). We suggest that PPNB lime plaster makers found new ways to improve binding properties and achieve a high quality standard by adding different materials to their lime plasters instead of producing more quicklime and slaking it until fully reacted, which requires more effort but does not necessarily result in better quality.

To the best of our knowledge, so far no evidence has been reported for the presence of lime plaster in PPNA sites in the southern Levant. It is therefore uncertain how exactly the evidence from NEG II relates to PPNB lime plaster technology more than 1000 years later. While, from a technological aspect there seems to be successive steps in lime plaster technological evolution, it is nevertheless possible that lime plaster technology was lost during the PPNA and reinvented during the PPNB. We hope that future studies of PPNA sites will help to clarify this question.

Lastly, although it is not within the scope of this article, the technology and production technique behind NEG II lime plaster may help, when integrated with other lines of evidence, to illuminate new aspects about how technological innovation during the end of the Epipalaeolithic was associated with communal effort, ritual activity and the rise of more complex forms of society. In addition, the increase in the scale of exploitation of natural resources, for instance the use of rocks required to produce lime plaster, could mark an important transition in the perception of the environment as part of a broader process of ‘domestication’ – one that goes beyond the mere domestication of plants and animals – the ‘domestication’ of the landscape. To conclude, the development of lime plaster technology forms an important aspect of human cultural evolution and deserves more attention when discussing the transformations in human society and culture during the Palaeolithic–Neolithic transition.
